# Synthesis and Self-Assembly of Poly(*N*-Vinylcaprolactam)-*b*-Poly(ε-Caprolactone) Block Copolymers via the Combination of RAFT/MADIX and Ring-Opening Polymerizations

**DOI:** 10.3390/polym12061252

**Published:** 2020-05-30

**Authors:** Rodolfo M. Moraes, Layde T. Carvalho, Gizelda M. Alves, Simone F. Medeiros, Elodie Bourgeat-Lami, Amilton M. Santos

**Affiliations:** 1 Laboratory of Polymers, Department of Chemical Engineering, Engineering School of Lorena, University of São Paulo, EEL-USP, Estrada Municipal do Campinho, s/n, P.O. Box 116, Lorena SP 12602-810, Brazil; rodolfommoraes@hotmail.com (R.M.M.); laydecarvalho@usp.br (L.T.C.); gizelda@dequi.eel.usp.br (G.M.A.); simonemedeiros@usp.br (S.F.M.); 2 Univ Lyon, Université Claude Bernard Lyon 1, CPE Lyon, CNRS UMR 5265, Chemistry, Catalysis, Polymers and Processes (C2P2), 43 Bvd. du 11 Novembre 1918, F-69616 Villeurbanne, France

**Keywords:** thermosensitive, amphiphilic, block copolymers, poly(*N*-vinylcaprolactam), poly(ε-caprolactone), ROP, RAFT/MADIX polymerization

## Abstract

Well-defined amphiphilic, biocompatible and partially biodegradable, thermo-responsive poly(*N*-vinylcaprolactam)-*b*-poly(ε-caprolactone) (PNVCL-*b*-PCL) block copolymers were synthesized by combining reversible addition-fragmentation chain transfer (RAFT) and ring-opening polymerizations (ROP). Poly(*N*-vinylcaprolactam) containing xanthate and hydroxyl end groups (X–PNVCL–OH) was first synthesized by RAFT/macromolecular design by the interchange of xanthates (RAFT/MADIX) polymerization of NVCL mediated by a chain transfer agent containing a hydroxyl function. The xanthate-end group was then removed from PNVCL by a radical-induced process. Finally, the hydroxyl end-capped PNVCL homopolymer was used as a macroinitiator in the ROP of ε-caprolactone (ε-CL) to obtain PNVCL-*b*-PCL block copolymers. These (co)polymers were characterized by Size Exclusion Chromatography (SEC), Fourier-Transform Infrared spectroscopy (FTIR), Proton Nuclear Magnetic Resonance spectroscopy (^1^H NMR), UV–vis and Differential Scanning Calorimetry (DSC) measurements. The critical micelle concentration (CMC) of the block copolymers in aqueous solution measured by the fluorescence probe technique decreased with increasing the length of the hydrophobic block. However, dynamic light scattering (DLS) demonstrated that the size of the micelles increased with increasing the proportion of hydrophobic segments. The morphology observed by cryo-TEM demonstrated that the micelles have a pointed-oval-shape. UV–vis and DLS analyses showed that these block copolymers have a temperature-responsive behavior with a lower critical solution temperature (LCST) that could be tuned by varying the block copolymer composition.

## 1. Introduction

Amphiphilic and stimuli-responsive block copolymers have been attracting an extensive scientific interest. Amphiphilic block copolymers are capable of self-assembling in aqueous solutions and, depending on their architecture, molar ratio, and chemical composition, ordered nanostructures of various morphologies including spherical micelles, cylindrical micelles, lamellae and vesicles, can be obtained [1]. This feature contributes to the potential applications of these materials in many fields including catalysis, microelectronics, nanoreactors and drug delivery systems [2,3].

On the other hand, stimulus-responsive polymers are macromolecules that undergo a change in their chemical or physical nature in response to an external stimulus (temperature, pH, electric field, light, etc.), which generally leads to changes in shape, conformation, hydrophilic/hydrophobic equilibrium or in the solubility of the polymer. Stimuli-responsive polymers have been used to incorporate and release active substances for controlled delivery systems [4]. Among all smart polymers, thermo-responsive polymers are frequently used because the temperature is an important physiological factor in the body, and some disease states manifest themselves by some changes in temperature [5]. In addition, hyperthermia techniques can be used with the intent of improving the targeting ability of the thermo-responsive carriers for the tissues [6].

Thermo-responsive polymers can be classified into two categories: polymers exhibiting a lower critical solution temperature (LCST) and those having an upper critical solution temperature (UCST). In the first case, the polymer (e.g., poly (*N*-vinylcaprolactam) (PNVCL), poly(*N*-vinylpyrrolidone) (PNVP), poly(*N*-vinylcarbazole) (PNK), or poly(*N*-isopropylacrylamide) (PNIPAAm)) is miscible with the solvent as long as the temperature of the solution is kept below the phase transition temperature, however, two immiscible phases are formed above this temperature: a diluted polymer phase and a concentrated polymer phase [7]. On the other hand, polymers exhibiting UCST show the opposite behavior, with a phase separation occurring upon cooling below the phase transition temperature [7]. However, there are also systems that exhibit both LCST and UCST [8]. Water-soluble thermo-responsive polymers change from hydrophilic to hydrophobic by heating the system above the transition temperature in the case of an LCST behavior and by cooling below the transition temperature in the case of UCST. In the two cases, the dehydration of the polymer phase at the transition temperature is due to polymer-polymer interactions becoming thermodynamically favored and it leads to a collapse of the polymer chains from elongated coils to collapsed globules [7]. Due to their unique responsive behavior, thermo-responsive polymers have been used in various fields besides controlled drug delivery, such as rheological control additives [9], gene delivery [10] thermal affinity separation [9], scaffolding for tissue regeneration [11], stabilization of colloids [12], oil–gas industry [13], thermally switchable optical devices [14] and bioimaging [15].

PNVCL is a thermosensitive polymer that has been extensively studied. This polymer has an LCST near the physiological temperature (35–38 °C) that varies as a function of molar mass and polymer concentration [16]. The LCST also depends on the chemical composition in the case of copolymers [16,17]. In addition, the biocompatibility, associated with the complexation ability with other molecules and the low toxicity of PNVCL, justify its use in several applications in the medical field [16,18].

Amphiphilic block copolymers containing poly(ε-caprolactone) (PCL) segments are very interesting in view of their pharmaceutical and biomedical applications due to their biocompatibility and biodegradability [19]. The degradation product of PCL is 6-hydroxyhexanoic acid, which is a naturally occurring metabolite in the human body [20]. Compared to other biodegradable aliphatic polyesters, PCL has several advantageous properties, including slow erosion kinetics, miscibility with various polymers and high permeability to small drug molecules [5]. However, the high crystallinity of this polymer decreases its compatibility with soft tissues and lowers its biodegradability [21]. Furthermore, PCL shows a lack of mechanical strength due to its low glass transition temperature (−60 °C) and low melting temperature (60 °C) [21]. These drawbacks can be overcome either by blending PCL with various polymers or preferably by the synthesis of PCL-based block copolymers [21].

The development of novel strategies to obtain block copolymers has attracted considerable attention, due to the possibility to combine specific properties found in each polymer segment separately. A wide variety of well-defined copolymers have been successfully synthesized by combining the use of the Reversible Addition-Fragmentation Chain Transfer (RAFT) and Ring-Opening Polymerization (ROP) techniques, as shown in the literature [22,23,24,25,26,27,28].

Reversible-deactivation radical polymerization (RDRP) techniques such as Atom Transfer Radical Polymerization (ATRP), RAFT and Nitroxide-Mediated Polymerization (NMP) have allowed the synthesis of various functional polymers with predetermined molar mass, narrow molar mass distribution, and numerous controlled architectures, from stars and branched polymers to block and graft copolymers. The living feature of RAFT polymerization is achieved through the reversible chain transfer of growing radicals to thiocarbonylthio compounds, such as dithioesters and trithiocarbonates. The RAFT process is very versatile and is suitable for the polymerization of most conjugated monomers [29]. However, RDRP of unconjugated monomers such as vinyl acetate (VAc), *N*-vinylcarbazole (NVK), *N*-vinylpyrrolidone (NVP) and *N*-vinylcaprolactam (NVCL) represents a challenge due to the high reactivity of the radical species formed during polymerization. For these types of monomers, the RAFT polymerization mediated by xanthates (dithiocarbonates) as chain transfer agents (RAFT/MADIX) is currently considered the most suitable [18].

On the other hand, the ROP technique can be applied to a wide range of monomers, with several types of initiators and catalytic systems [30]. This polymerization technique can be driven by different mechanisms: anionic or cationic polymerization and coordination-insertion mechanism. In addition, the ROP technique has enabled the synthesis of block copolymers with the molar mass and molar mass distribution controlled using hydroxylated macrochains during the cyclic monomer ring-opening polymerization. Konishcheva et al. [31], Ponjavic et al. [32], Ali et al. [33] and Kheiri Manjili et al. [34], for example, used poly(ethylene oxide) monohydroxylated homopolymers as macroinitiators in the ring-opening polymerization of ε-caprolactone (ε-CL), using Sn(Oct)_2_ as the catalyst. This artifice allowed to pre-establish the length of the PCL block and resulted in the synthesis of poly(ethylene oxide)-b-poly(ε-caprolactone) (PEO-*b*-PCL) block copolymers with narrow molar mass distributions (Đ < 1.5). On the other hand, Choi et al. [35] and Wu et al. [36] performed the ROP of ε-CL using hydroxyl-terminated PNIPAAm or PNVCL homopolymers, respectively, previously synthesized by radical polymerization of NIPAAm or NVCL in the presence of 2-mercaptoethanol as chain transfer agent (CTA).

In this context, amphiphilic, biocompatible and partially biodegradable, thermo-responsive PCL-*b*-PNVP, PCL-*b*-PNVCL and poly(*D,L*-lactide)-*b*-PNVP (PDLLA-*b*-PNVP) diblock copolymers, for example, were successfully synthesized by Mishra et al. [22], Yu et al. [23] and Ramesh et al. [24], respectively, by combining ROP and the RAFT/MADIX technique. Hydroxyl-terminated PCL or PDLLA polymers were first synthesized by ROP, and their ω chain end was then converted to an *O*-ethyl xanthate end group for the RAFT/MADIX polymerization of pre-established unconjugated *N*-vinyl monomers. Kang et al. [25] also reported the preparation of PCL-*b*-PNVP block copolymers by these two polymerization techniques. These authors used a hydroxyl-functionalized xanthate RAFT agent, 2-hydroxyethyl 2-(ethoxycarbonothioylthio) propanoate (HECP), as a dual initiator for ROP and RAFT polymerization in a one-pot procedure. The PCL block was first synthesized by ROP of ε-CL using diphenyl phosphate as a catalyst followed by RAFT polymerization of NVP at 30 °C, using 2,2′-azobis (4-methoxy-2,4-dimethylvaleronitrile) (V-70) as initiator. Finally, in this last example, Yu et al. [26] showed the synthesis of three different block copolymers, PCL-*b*-PNVCL, poly(*δ*-valerolactone)-*b*-PNVCL (PVL-*b*-PNVCL) and poly(trimethylene carbonate)-*b*-PNVCL (PTMC-*b*-PNVCL), using a similar one-pot synthetic route to the above-mentioned. The hydrophobic blocks were first synthesized by ROP of cyclic monomers in the presence of HECP, and the RAFT/MADIX polymerization of the PNVCL block was followed by adding NVCL and V-70 to the reaction mixture.

Aiming at combining the hydrophilicity, biocompatibility and thermosensitive properties of PNVCL with the hydrophobicity and biodegradability of PCL, we performed the synthesis of well-defined PNVCL-*b*-PCL block copolymers via a novel synthetic route which consists in a combination of the RAFT/MADIX and ROP techniques. In order to achieve well-defined sizes of each segment of the block copolymer, this synthetic process was carried out in two-steps. First, an hydroxyl end-capped PNVCL homopolymer comprising a xanthate group (X–PNVCL–OH) was obtained by NVCL RAFT/MADIX polymerization mediated by a previously synthesized xanthate-based CTA: *O*-ethyl S-4-(hydroxymethyl) benzyl carbonodithioate. The PNVCL xanthate end group was removed by a radical-induced process using a large excess of 2,2′-azobis(isobutyronitrile) (AIBN). PNVCL-*b*-PCL block copolymers were then obtained by ROP of ε-CL using hydroxyl end-capped PNVCL homopolymer as the macroinitiator. These (co)polymers were characterized by Size Exclusion Chromatography (SEC), Fourier-Transform Infrared spectroscopy (FTIR), Proton Nuclear Magnetic Resonance spectroscopy (^1^H NMR) and Differential Scanning Calorimetry (DSC) measurements. Finally, PNVCL-*b*-PCL micelles were prepared by the nanoprecipitation method, and their physicochemical and thermosensitive properties were investigated.

## 2. Materials and Methods

### 2.1. Materials

*N*-vinylcaprolactam (NVCL, kindly supplied by BASF) was distilled under reduced pressure. ε-caprolactone (ε-CL, 97%, Aldrich) was dried over CaH_2_ and distilled under reduced pressure before use. AIBN was recrystallized from ethanol, and stored at 4 °C. Toluene (99.5%, Synth), triethylamine (TEA, 99%, Vetec), tetrahydrofuran (THF, 99%, Synth) and 1,4-dioxane (99%, Synth) were dried and fractionally distilled from sodium. Ethanol (95% Synth) was dried and fractionally distilled from calcium hydride. Stannous octoate (Sn(Oct)_2_, 92.5–100%, Aldrich), 1,3,5-trioxane (≥99%, Aldrich), 4-(chloromethyl)phenyl methanol (CMPM, 99%, Aldrich), potassium ethyl xanthogenate (KEX, 96%, Aldrich), magnesium sulfate (MgSO_4_ XH_2_O, 98%, Vetec), pyrene (98%, Aldrich), dichloromethane (DCM, 99.5%, Synth), diethyl ether (98%, Synth) and acetone (99.5%, Synth) were used as received.

### 2.2. Methods

#### 2.2.1. Synthesis of Hydroxyl-Terminated Poly(N-Vinylcaprolactam) (PNVCL–OH)

**Scheme 1:**
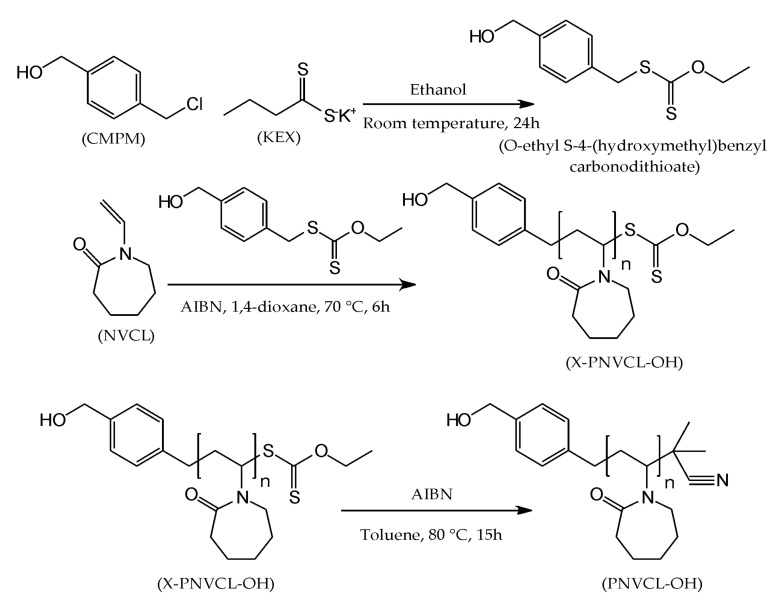
Synthetic routes toward *O*-ethyl S-4-(hydroxymethyl)benzyl carbonodithioate and poly(*N*-vinylcaprolactam) (PNVCL–OH) homopolymer, and the procedure used for xanthate end group removal from the PNVCL homopolymer.

PNVCL–OH was synthesized by RAFT/MADIX polymerization of NVCL monomer using *O*-ethyl *S*-4-(hydroxymethyl) benzyl carbonodithioate as the chain transfer agent, followed by xanthate end group removal (Scheme 1).

##### Synthesis of the O-Ethyl S-4-(Hydroxymethyl) Benzyl Carbonodithioate Chain Transfer Agent

The *O*-ethyl S-4-(hydroxymethyl) benzyl carbonodithioate was prepared and extracted according to a methodology adapted from Binauld et al. [37]. In a 100 mL round-bottom flask, a solution of 4-(chloromethyl) benzyl alcohol (4.904 g, 31.0 mmol) in 20 mL of dry ethanol was prepared with magnetic stirring under nitrogen atmosphere. Then, a solution of KEX (7.764 g, 46.5 mmol) in 70 mL of dry ethanol was added dropwise to the previous solution maintaining stirring under nitrogen using a Schlenk line apparatus. The reaction mixture was stirred for an additional 24 h at room temperature. The ethanol was evaporated under vacuum at room temperature, then, the concentrated mixture was dissolved in 50 mL of DCM. This mixture was washed several times with water (10 × 20 mL). The organic phase was dried over magnesium sulfate and then under vacuum at room temperature, yielding a white powder (yield = 93.9%). The ^1^H NMR spectrum of *O*-ethyl S-4-(hydroxymethyl)benzyl carbonodithioate is shown in Figure S1 (Supplementary Materials). ^1^H NMR (ppm, CDCl_3_), δ: 1.42 (t, 3H, CH_3_), 4.36 (s, 2H, SCH_2_), 4.65 (q, 2H, CH_3_CH_2_), 4.68 (d, 2H, CH_2_OH), 7.29–7.37 (m, 4H, Ph).

##### RAFT/MADIX Polymerization of NVCL

Hydroxyl-terminated PNVCL was synthesized by RAFT/MADIX polymerization of NVCL mediated by *O*-ethyl S-4-(hydroxymethyl)benzyl carbonodithioate as the chain transfer agent, at 70 °C using AIBN as the initiator and 1,4-dioxane as the solvent. This reaction was performed with the [NVCL]_0_:[CTA]_0_:[AIBN]_0_ feed molar ratio = 150:1:0.1. In a dried and nitrogen purged round-bottom flask, 58.0 mg (0.24 mmol) of CTA, 5 g (35.91 mmol) of NVCL, 3.9 mg (0.024 mmol) of AIBN and 101.1 mg (1.12 mmol) of 1,3,5-trioxane (internal standard) were dissolved in 2.5 mL of 1,4-dioxane. A homogeneous solution was obtained after stirring, and degassed under nitrogen for 30 min using a Schlenk line apparatus. After that, the flask was immersed in an oil bath at 70 °C for 12 h. In order to optimize the NVCL RAFT/MADIX polymerization, the NVCL polymerization rate, and the evolution of the number average molar mass (*M*_n_) and the dispersity (Đ) of the polymer was monitored by ^1^H NMR and HPLC, respectively. After that, another RAFT/MADIX polymerization reaction was carried out in the same conditions, but on a larger scale (all quantities were increased by a factor of 6) and for only 6 h. This second reaction was stopped by cooling the reaction mixture in an ice bath. The resulting polymer was purified by precipitation in cold diethyl ether, recovered by filtration, dried under vacuum at room temperature for 24 h, and isolated as a white powder. The precipitation procedure was repeated three times.

##### Xanthate End Group Removal from PNVCL Homopolymer by a Radical-Induced Process

Removal of the xanthate end group by radical-addition-fragmentation-coupling from PNVCL homopolymer previously synthesized by RAFT/MADX polymerization, was carried out using a large excess of AIBN ([AIBN]_0_:[X–PNVCL–OH]_0_ feed molar ratio = 30:1) following a method previously reported by Perrier et al. [38]. A mixture of hydroxyl end-capped PNVCL homopolymer comprising a xanthate group (X–PNVCL–OH, 6.0 g, 0.94 mmol), AIBN (4.633 g, 28.2 mmol) and toluene (60 mL) was introduced in a 150 mL round-bottom flask. The mixture was stirred and degassed under nitrogen for 45 min using a Schlenk line equipment. Then, the flask was immersed in an oil bath at 80 °C for 15 h. After this period, the polymer was purified and isolated as described above for PNVCL homopolymer.

#### 2.2.2. Ɛ-CL Ring-Opening Polymerization Initiated by PNVCL–OH Homopolymer

**Scheme 2:**
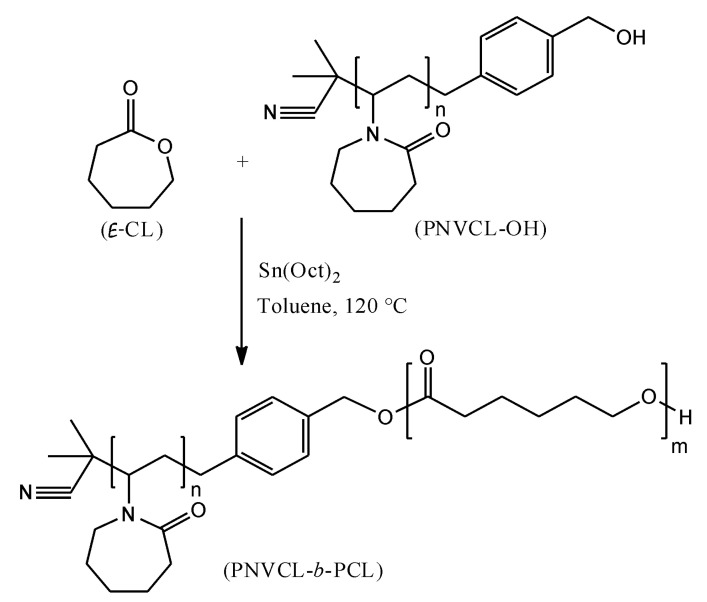
Synthetic route of amphiphilic PNVCL-*b*-PCL block copolymers.

PNVCL-*b*-PCL block copolymers were synthesized by ring-opening polymerization of ε-CL initiated by PNVCL–OH homopolymer with Sn(Oct)_2_ as the catalyst (Scheme 2). Two different ε-CL molar concentrations with respect to PNVCL–OH ([ε-CL]_0_:[PNVCL–OH]_0_ molar ratio = 31.25 and 62.50) were used. 1.6 or 0.8 g of PNVCL–OH was added in a flask equipped with a magnetic stirring bar under nitrogen at 120 °C for 15 h, in order to remove any traces of water that could be present in the polymer. After cooling the flask to room temperature, 46.9 mg (0.12 mmol) of catalyst, 0.881 g (7.7 mmol) of ε-CL and 5.0 or 3.4 mL of toluene were successively added to the flask. Thereafter, the mixture was kept under stirring under nitrogen at room temperature for 30 min. The flask was subsequently sealed with a silicone septum, and the polymerization reaction was performed in an oil bath at 120 °C under stirring. The ε-CL polymerization rate was monitored by ^1^H NMR, and the mixture was allowed to react until monomer conversion was close to 95%. Lastly, the copolymer was purified and isolated as reported above.

#### 2.2.3. Micelles Preparation

The micelles were prepared by applying the nanoprecipitation technique (also known as the solvent displacement method) following a procedure reported in the literature [39]. Typically, 50 mg of the copolymer was dissolved in 2.5 mL of THF. Then, the copolymer solution was added dropwise into 10 mL of ultrapure water at 20 °C, and THF was removed using a rotary evaporator at 20 °C for 2 h. The obtained solution was transferred into a 25 mL volumetric flask, followed by dilution to the calibration mark with ultrapure water in order to obtain 2 mg mL^−1^ micelles.

### 2.3. Characterizations

The number average molar masses (*M*_n_) and dispersities (Đ) were determined by SEC (Waters 1515) using THF with triethylamine (0.3% v/v) as the solvent, at 35 °C with a flow rate of 1.0 mL min^−1^ on three Phenogel columns (10^3^, 10^4^ and 10^6^ Å) connected in series to a 2414 differential refractive index detector. A high-performance liquid chromatography (HPLC, Shimadzu, Kyoto, Japan) was also used to determine the *M*_n_ and Ð using THF with triethylamine (0.3% v/v) as the solvent, at 35 °C with a flow rate of 0.8 mL min^−1^ on two Phenogel columns (10^4^ and 10^6^ Å) connected in series to a differential refractive index detector (Sil−20A Shimadzu). The columns were calibrated using polystyrene (PS) standards. The ^1^H NMR spectra were obtained in a Varian Mercury-300 NMR spectrometer (300 MHz) at room temperature using deuterated dimethylsulfoxide (DMSO-*d*_6_) or chloroform (CDCl_3_) as solvents, and the chemical shifts are reported in parts per million (ppm) using tetramethylsilane (TMS) as the internal standard. The molar masses of PNVCL homopolymers were determined by ^1^H NMR by comparing the integration area of the methane peaks of the PNVCL backbone at 4.30 ppm (1H) with the one of the methane peaks of the aromatic ring present at the end of the PNVCL chain at 7.2 ppm (4H). The molar masses of block copolymers (BCP) were determined by ^1^H NMR using the following method: *M*_n NMR (BCP)_ = *M*_n NMR (PNVCL segment)_ + *M*_n NMR (PCL segment)_, where *M*_n NMR (PNVCL segment)_ = *M*_n NMR (PNVCL-OH)_ = 6480 g mol^−1^, and the *M*_n NMR (PCL segment)_ was determined by comparing the integrated area of the methylene peak for the PCL block at 4.05 ppm (2H), with that of the methylene peak adjacent to the hydroxyl group present at the end of the PCL block at 3.64 ppm (2H). FTIR spectra were collected using a Shimadzu IRPrestige-21 spectrometer using the KBr disk method. Absorbance measurements of the polymer organic solutions in THF were performed on a Thermo Scientific Genesys *10UV* spectrophotometer, in the 190 to 1100 nm range. The (co)polymers thermal properties were evaluated using a *TA* Instruments Q20 DSC under nitrogen atmosphere. The instrument was calibrated with indium before use. The samples were first heated from −80 to 180 °C at a 10 °C min^−1^ heating rate, followed by quenching to −80 °C. Then, the samples were re-heated to 220 °C at a rate of 10 °C min^−1^. The critical micelle concentrations (CMC) of the block copolymers were determined by fluorescence measurements using pyrene as a probe. A pyrene solution in acetone was added into a series of volumetric flasks in such an amount that the final concentration of pyrene in each solution was 6.10^−7^ mol L^−1^. The acetone was then allowed to completely evaporate. A copolymer solution was added into the volumetric flasks and diluted until the calibration mark using deionized water in order to obtain different copolymer concentrations ranging from 5.10^−5^ to 0.05 mg mL^−1^. The samples were stored at room temperature overnight to equilibrate micelles and pyrene. Steady-state fluorescence excitation spectra were recorded on a Varian Cary Eclipse fluorescence spectrophotometer at 390 nm emission wavelength. The hydrodynamic average diameter (Z_av._) and the size distribution (PdI, the smaller this value, the lower the distribution) of the micelles were determined by dynamic light scattering (DLS) using a Malvern Nano ZS instrument. The LCST of the homopolymers and of the thermosensitive micelles in aqueous solutions was determined using a Thermo Scientific Genesys 10UV spectrophotometer equipped with a Thermo Fisher Scientific air-cooled temperature controller. The transmittance of aqueous solutions of the polymer at ʎ = 500 nm was recorded in a 1.0 cm path length quartz cell. The rate of heating was set at 1 °C min^−1^ with hold steps of 5 min at each temperature. Values for the LCST of aqueous solutions of the polymers were determined at a temperature with half of the optical transmittance between below and above transitions. Cryogenic transmission electron microscopy (cryo-TEM) images of the micelles were obtained using a Philips CM120 microscope from the Centre Technologique des Microstructures (CTµ), platform of the Université Claude Bernard Lyon 1, in Villeurbanne, France. The diluted samples were dropped onto 300 Mesh holey carbon films (Quantifoil R2/1), and quench-frozen in liquid ethane. Samples were transferred in the microscope using a precooled Gatan 626 specimen holder, and observed at an accelerating voltage of 120 kV.

## 3. Results

### 3.1. Synthesis of Hydroxyl-Terminated PNVCL Homopolymers (PNVCL–OH)

The chemical route used for the synthesis of the PCL-*b*-PNVCL block copolymers is shown in Scheme 1. The block copolymers were synthesized via a combination of RAFT/MADIX and ring-opening polymerizations. In the first step, the *O*-ethyl S-4-(hydroxymethyl)benzyl carbonodithioate xanthate chain transfer agent was synthesized and used to mediate the RAFT/MADIX polymerization of NVCL to give rise to well-defined hydroxyl-terminated PNVCL homopolymers comprising a xanthate group (X–PNVCL–OH). Although the number-average molar mass (*M*_n_) values of the polymer increased linearly with conversion, the molar mass dispersity (Ð) also slightly increased with time (Supplementary Materials, Figure S2), suggesting that side reactions occurred under our experimental conditions, which may be due to the low thermal stability of the xanthate end group. During the RAFT/MADIX polymerization of the poly(*N*-vinylpyrrolidone) (PNVP) mediated by the same CTA, *O*-ethyl S-4-(hydroxymethyl)benzyl carbonodithioate, Binauld et al. [37] observed significant thermal degradation of the *O*-ethyl xanthate chain end, especially when high temperatures and/or reaction times were used. From the optimization of the NVCL RAFT/MADIX polymerization conditions at 70 °C, using AIBN as initiator, it appeared that in order to keep the polymer dispersity below 1.5, the reaction time had to be limited to about 6 h (NVCL conversion ≈ 34%). Thereafter, in order to use the hydroxylated PNVCL as a macroinitiator in the ε-CL ring-opening polymerization at high temperature (120 °C), the xanthate group present at the end of the homopolymer chain was removed by radical-addition-fragmentation-coupling reaction using an excess of AIBN. Due to the high reactivity and low thermal stability of the xanthate, this chemical group was removed aiming to reduce the probability of forming undesirable products during the synthesis of PNVCL-*b*-PCL block copolymers.

Perrier et al. [38] have reported the removal of dithiobenzoate end groups from poly(methyl methacrylate) (PMMA) by reacting the polymer with an excess of AIBN in toluene to prepare PMMA chains bearing cyanoisopropyl end groups. This process was also successfully applied for the removal of various thiocarbonylthio end groups from polystyrene (PS), poly(methyl acrylate) (PMA) and other acrylic polymers [38]. A similar strategy was also applied to PS bearing butyl trithiocarbonate end groups giving around 95% of end group removal [40]. It is important to mention that a large excess of the initiator (ca. 20-fold excess, typically AIBN or azobis(cyclohexanenitrile) (ACHN)) must be used to avoid by-products formation by termination reactions between the propagating radicals of the macro-RAFT agent. In a similar experiment with PMMA and a smaller excess of the initiator (10-fold), for example, unsaturated end groups from termination by disproportionation, were observed [38]. In our work, we used a [AIBN]_0_:[X–PNVCL–OH]_0_ molar ratio of 30:1, as previously mentioned.

**Figure 1:**
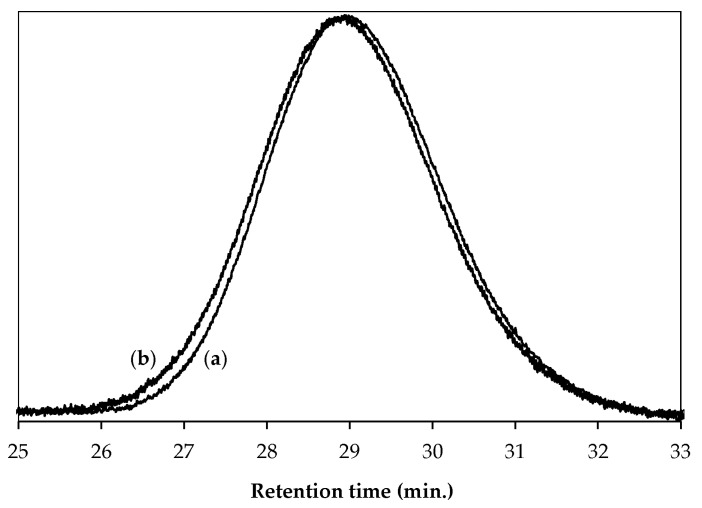
Size exclusion chromatography (SEC) chromatogram of PNVCL homopolymer before ((**a**), X–PNVCL–OH) and after ((**b**), PNVCL–OH) chain end removal by reaction with 2,2′-azobis (isobutyronitrile) (AIBN).

Figure 1 shows the SEC chromatogram obtained for the PNVCL homopolymer before (X–PNVCL–OH) and after (PNVCL–OH) reaction with AIBN. The number average molar mass (*M*_n_) and dispersity (Ð) values obtained for these polymers are presented in Table 1.

The experimental molar mass determined by NMR (*M*_n NMR_) of the hydroxyl-terminated PNVCL homopolymer comprising xanthate group (X–PNVCL–OH) was in good agreement with the theoretical molar mass (*M*_n theo_) (Table 1). However, there was a significant discrepancy between them and the molar mass obtained by SEC (*M*_n SEC_). The reason for this discrepancy lies in the use of a polystyrene calibration that is not fully appropriate in this case. Nevertheless, the sample showed a low dispersity value (Đ < 1.5), as desired.

As mentioned above, during the thiocarbonylthio end group removal process by the radical coupling technique, termination reactions may occur by combining the polymer radicals, which may result in the formation of a polymer with a higher molar mass and/or a higher dispersity than the starting polymer. However, from the SEC chromatograms shown in Figure 1, and from the *M*_n_ and Ð values shown in Table 1, it can be noted that X-PNVCL–OH and PNVCL–OH homopolymers exhibit monomodal molar mass distributions, and similar values of *M*_n SEC_ and Ð. Therefore, these results indicate that there was no occurrence of this type of secondary reaction during the removal reaction of the xanthate group present at the end of the PNVCL chains.

**Figure 2:**
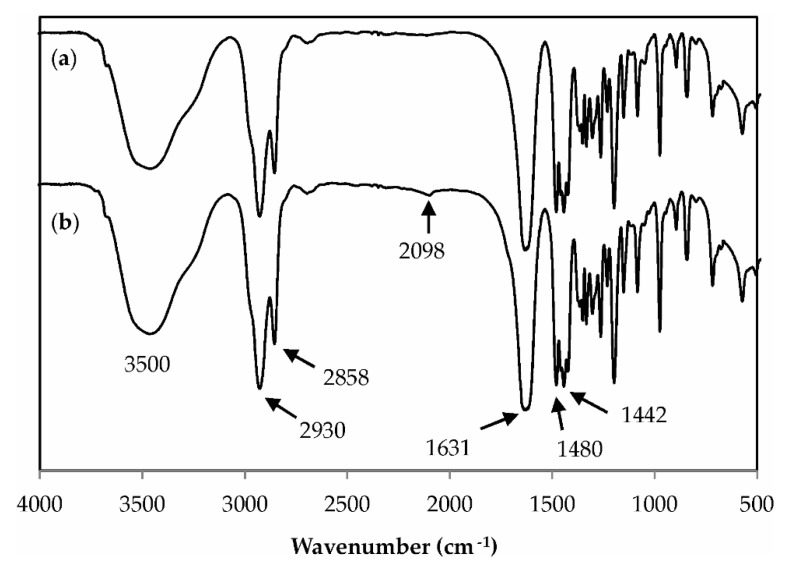
Fourier-Transform Infrared (FTIR) spectra of PNVCL–OH before ((**a**), X-PNVCL–OH) and after ((**b**), PNVCL–OH) reaction with AIBN.

The FTIR spectra of the PNVCL homopolymers before (X-PNVCL–OH) and after (PNVCL–OH) reaction with AIBN are presented in Figure 2. Both polymers show a strong absorption at 1650 cm^−1^ assigned to the axial deformation of the carbonyl (C=O) of the caprolactam ring. The peaks at 2930 cm^−1^ and 2858 cm^−1^ are due to C–H stretching, while the absorption bands at 1442 cm^−1^ and 1480 cm^−1^ can be assigned respectively to the -CH_2_- angular and the C–N axial deformations. The shoulder at 3274 cm^−1^ is assigned to N-H stretching vibration. In addition, the broad absorption band in the region 3200–3600 cm^−1^ is due to hydroxyl groups, which may arise from the CTA–OH leaving group present at the end of the polymer chain, or from the moisture adsorbed by the sample during the analysis. In contrast to X–PNVCL–OH, the FTIR spectrum of PNVCL–OH exhibits a small intensity band at 2098 cm^−1^, corresponding to the axial deformation of the CΞN nitrile group from AIBN.

The ^1^H NMR spectra of X-PNVCL–OH and PNVCL–OH in DMSO-*d*_6_ are depicted in Figure S3 (Supplementary Materials). Note that both samples gave the typical signals of PNVCL at δ (ppm) = 4.35 (1H, –NCH– α position, **c**), 3.05 (2H, –NCH_2_–, **d**), 2.25 (2H, –C(=O)CH_2_–, **h**) and 1.0–2.0 (6H, –CH_2_–, **e** and **g** of the caprolactam ring, and 2H, –CH_2_– of the backbone chain of the polymer, **i**). The peaks located at about 7.1 ppm (4H, =CH– of the aromatic ring, **k**) and 4.4 ppm (2H, –CH_2_OH, **l**) refer to the protons of the methine groups of the aromatic ring, and the methylene group of the CTA, respectively. In addition, by comparing the spectrum of PNVCL–OH with that of X–PNVCL–OH, it was possible to observe a significant reduction in the characteristic peak intensity of the methylene protons (2H, –CH_2_CH_3_, **b**) of the xanthate group at 4.6 ppm indicating successful removal of this functional group. However, the characteristic chemical shift of the methyl protons (3H, –CH_3_, **a’** and **b’**) of the isobutyronitrile group could not be observed in the spectrum of PNVCL–OH, since this peak overlaps with the PNVCL peaks (**e**, **f**, **g** and **i**).

**Figure 3:**
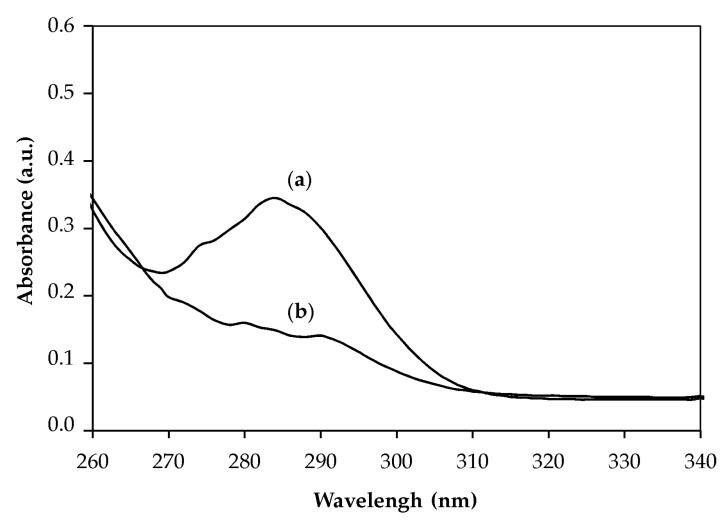
UV–vis spectra of the PNVCL macroinitiator before ((**a**), X–PNVCL–OH) and after ((**b**), PNVCL–OH) reaction with AIBN.

From the UV/vis spectra of X-PNVCL–OH and PNVCL–OH (Figure 3) it was possible to observe a significant reduction in the intensity of the characteristic absorption band of the C=S group, at 265–310 nm, for PNVCL–OH, in comparison to X–PNVCL–OH. Therefore, this result indicates a high efficiency in the methodology used to remove the xanthate group from the PNVCL chains. However, the low-intensity band observed in the spectrum of PNVCL–OH indicates that the xanthate group removal was not complete.

### 3.2. ε-CL Ring-Opening Polymerization Initiated by PNVCL–OH Homopolymer

**Figure 4:**
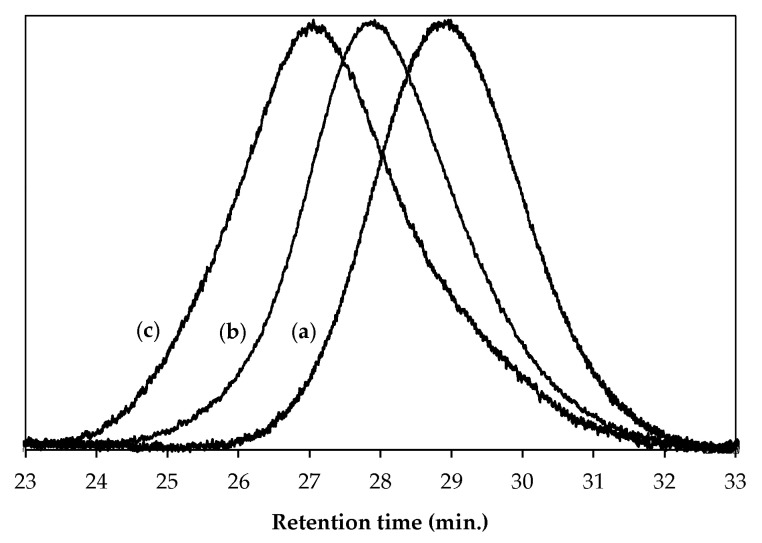
SEC chromatograms of (**a**) PNVCL–OH, (**b**) PNVCL-*b*-PCL (1) and (**c**) PNVCL-*b*-PCL (2).

In order to synthesize PNVCL-*b*-PCL block copolymers, the hydroxyl-terminated poly(*N*-vinylcaprolactam) (PNVCL–OH) was used as macroinitiator in the ε-CL ring-opening polymerization. Two different ε-CL molar concentrations relative to PNVCL–OH ([ε-CL ]_0_:[PNVCL–OH]_0_ molar ratio = 31.25 and 62.50) were used to obtain block copolymers with the same PNVCL hydrophilic block length, but with two different PCL hydrophobic block lengths (hereafter labeled PNVCL-*b*-PCL (1) and PNVCL-*b*-PCL (2), respectively). The highest molar ratio was selected so that both PNVCL and PCL blocks had similar lengths. On the other hand, the lowest molar ratio was selected to prepare a copolymer with the PCL block length significantly smaller than that of the PNVCL block. The SEC chromatograms of PNVCL–OH, PNVCL-*b*-PCL (1) and PNVCL-*b*-PCL (2) are shown in Figure 4. Table 2 shows the conversion values of ε-CL, and the molar masses (*M*_n_) and dispersity (Ð) values of the copolymers.

As seen in Figure 4, both block copolymers exhibit a unimodal molar mass distribution, a relatively low dispersity and molar masses significantly higher than the one of the PNVCL–OH homopolymer. These results indicate successful extension of the PNVCL chains, and the formation of a block copolymer. Besides, there is no residual PNVCL–OH and/or PCL homopolymer in the reaction product.

**Figure 5:**
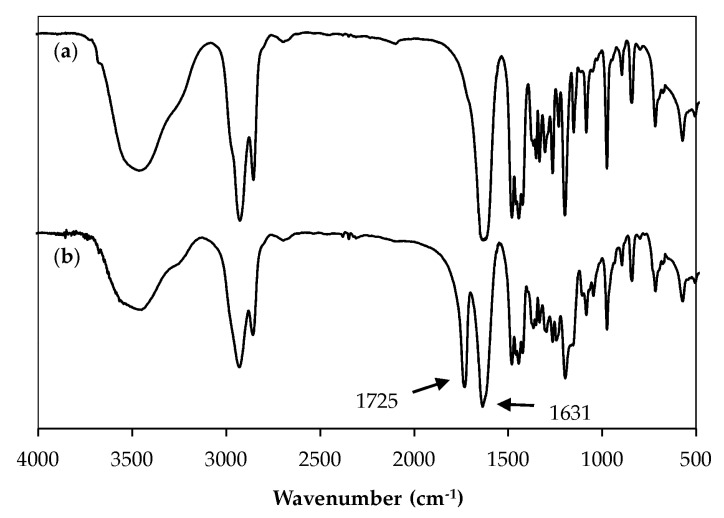
FTIR spectra of (**a**) PNVCL–OH and (**b**) PNVCL-*b*-PCL (1).

The FTIR spectra of PNVCL–OH and PNVCL-*b*-PCL (1) are depicted in Figure 5. As expected, compared to PNVCL–OH, the spectrum of the block copolymer, in addition to the deformation bands characteristic of the PNVCL, clearly shows an intense vibration band at 1725 cm^−1^ corresponding to the C=O vibrations of the aliphatic esters of PCL, confirming the formation of the PCL segment.

In addition, the ^1^H NMR spectrum of the block copolymer in CDCl_3_ displays all characteristic peaks of PNVCL and PCL segments (Figure S4, Supplementary Materials). The characteristic peaks of PCL present in these spectra are δ (ppm): 4.05 (2H, –CH_2_OC(=O)-, **p**), 3.64 (2H, –CH_2_OH, **p’**), 2.30 (2H, –OC(=O)CH_2_-, **m**), 1.64 and 1.37 (2H, -CH_2_- of the aliphatic carbon chain, **n** and **o**, respectively). Moreover, by comparing the spectrum of the PNVCL–OH with that of the copolymer, the PNVCL chain extension by ε-CL ring-opening polymerization was confirmed from the displacement of the methylene protons at 4.63 ppm (2H, -CH_2_OH, **l**) to 5.05 ppm (2H, -CH_2_OC(=O)-, **l**).

**Figure 6:**
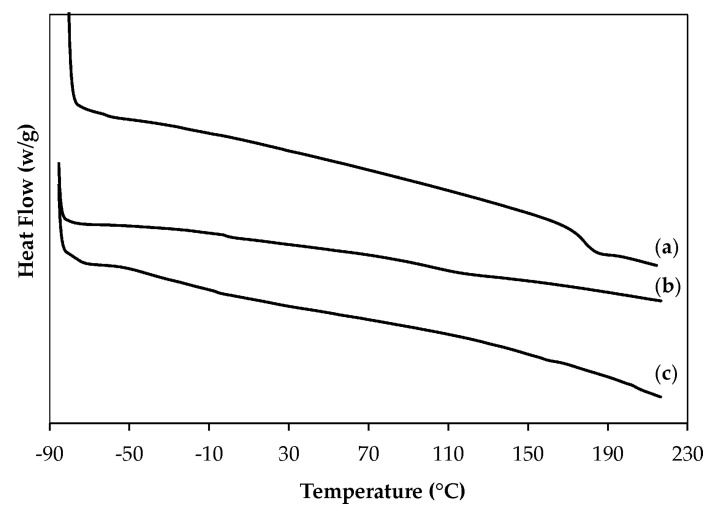
Differential Scanning Calorimetry (DSC) thermograms of (**a**) PNVCL–OH, (**b**) PNVCL-*b*-PCL (1) and (**c**) PNVCL-*b*-PCL (2).

PNVCL–OH and the PNVCL-*b*-PCL block copolymers were next characterized by DSC, as shown in Figure 6. PNVCL is an amorphous polymer [41], with a glass transition temperature (T_g_) of around 147 °C [42,43,44]. However, it is well known that its T_g_ can be influenced by several factors such as molar mass, dispersity, purity [45], and the presence of water in the polymer [43,44]. In our work, the determined T_g_ value of the dried PNVCL–OH was 177.2 °C, which is close to the T_g_ values reported by Usanmaz et al. [46] and Durkut et al. [47], (i.e., 174.6 and 174.0 °C, respectively). In the literature, the T_g_ and melting point temperatures (T_m_) of the PCL homopolymer have been reported to be about −60.7 °C and 56.8 °C, respectively [48,49]. The DSC thermograms of the PNVCL-*b-*PCL (1) did not show any event that could be attributed to thermal properties related to PCL, such as T_g_. This is probably due to the short length of the PCL block, PNVCL being the dominant phase in this case. However, this event was observed at −30.5 °C for PNVCL-*b*-PCL (2) due to the increase of the PCL block length. A T_g_ around 105.0 °C was observed for PNVCL-*b*-PCL (1); that can be attributed to the T_g_ of the PNVCL block, which was shifted toward a lower temperature due to the plasticizing effect of the PCL block [50]. However, this event (i.e., the T_g_ of the PNVCL segment) was not observed for PNVCL-*b*-PCL (2) due to the PCL being the dominant phase and/or due to the plasticizing effect of the PCL block being more pronounced for this copolymer that contains a longer PCL segment, or finally due to the analysis conditions. In addition, the absence of T_m_ peak in the block copolymer thermograms suggests that both materials have a low degree of crystallinity or exhibit amorphous polymer characteristics, since the PNVCL segment covalently bound to the PCL segment must have restricted the PCL crystallization, compromising the regularity of their crystalline structure.

### 3.3. Determination of the CMC of the Block Copolymers

**Figure 7:**
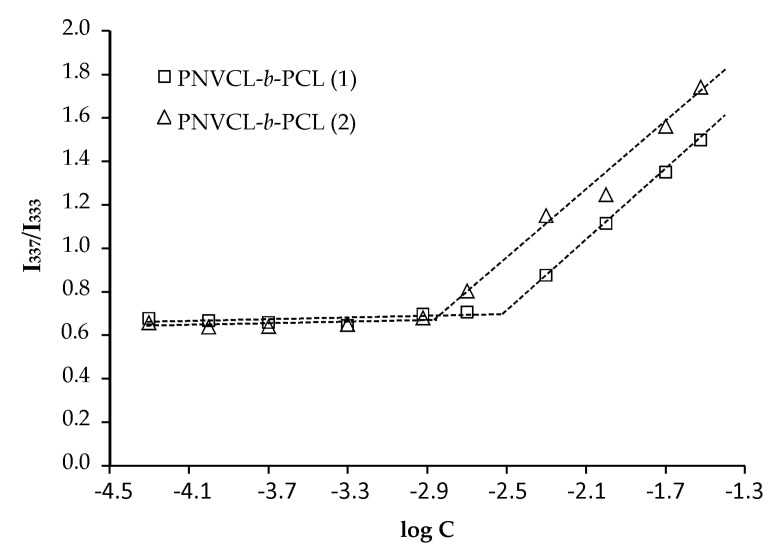
Plots of I_337_/I_333_ versus the logarithm of the PNVCL-*b*-PCL block copolymers concentrations and determination of the critical micelle concentrations (CMC) of the copolymers.

The CMC of the PNVCL-*b*-PCL block copolymers was determined using a fluorescence technique with pyrene as the fluorescence probe. Figure 7 shows the plot of the I337/I333 intensity ratio (from fluorescence measurement) versus the logarithm of the corresponding concentration of the copolymer in water (in mg mL^−1^). At a certain concentration, the intensity ratio started to increase dramatically. This increase reflects the incorporation of pyrene into the hydrophobic core of the micelles. The intersection of two straight lines: the baseline and the rapidly rising I337/I333 line is considered as the CMC of the amphiphilic block copolymer [19]. From this plot, CMC values of 2.9 10^−3^ and 1.4 10^−3^ mg mL^−1^ were obtained for PNVCL-*b*-PCL (1) and PNVCL-*b*-PCL (2), respectively. As seen in Table 2, the CMC decreased as the length of the hydrophobic block increased, in agreement with the literature [5,19,36]. This result was expected since a higher length of the hydrophobic segment results in stronger interactions between the hydrophobic chains; therefore, a lower concentration of polymer in water is necessary to induce micellization.

### 3.4. Micelles Morphology and Effect of the Hydrophobic Block Length on the Hydrodynamic Diameter of the Polymeric Micelles

Aiming to evaluate the effect of the hydrophobic block length on the average size of PNVCL-*b*-PCL micelles, micellar solutions were prepared via nanoprecipitation of PNVCL-*b*-PCL block copolymers, and then analyzed by DLS. The hydrodynamic diameter (Z_av_) of the micelles formed from PNVCL-*b*-PCL (1) and PNVCL-*b*-PCL (2) (2 mg mL^−1^) was about 86 nm and 117 nm, respectively. In addition, both PNVCL-*b*-PCL micelles exhibited a monomodal size distribution (Figure S5, Supplementary Materials) and PdI values of around 0.22 (Table 2). These Z_av_ values are close to the values reported by Wu et al. [5] and Wu et al. [19] for micelles prepared from PNVCL-*b*-PCL using a similar approach as the one reported in this work. As expected, the diameter of the micelles decreased with the increase of PCL length, thus suggesting that the size of the micelle could be adjusted by changing the length of the hydrophobic block in the copolymer. These results are in agreement with the results reported in the literature for amphiphilic block copolymers [5,19,28,36].

**Figure 8:**
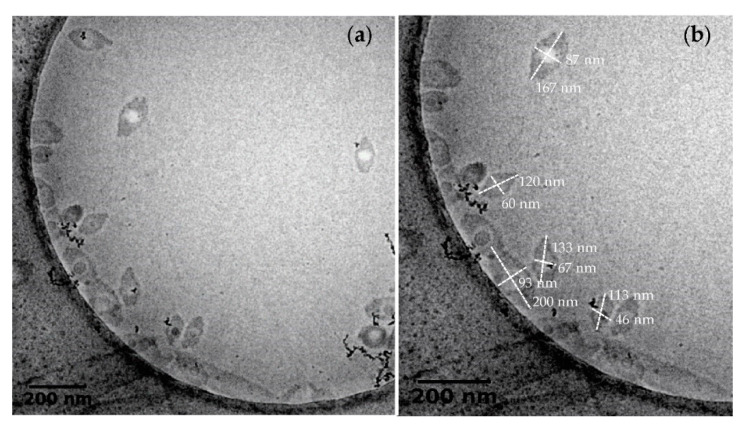
(**a**,**b**) Cryo-TEM micrographs of PNVCL-*b*-PCL (1) micelles.

The morphology of the PNVCL-*b*-PCL (1) micelles, as visualized by cryo-TEM, are shown in Figure 8. As seen in the Figure, the copolymers aggregated into pointed-oval-shape micelles consisting of a hydrophilic shell around the micellar hydrophobic core (Figure 8a). As expected, these oval shape nanostructures show two distinct diameter values, one referring to the largest diameter and the other relative to the smallest diameter. This may be also inferred from the relatively broad size distribution of these nanoparticles (Figure S5, Supplementary Materials). It appears that the largest diameter value varies from 113 to 200 nm, while the smallest diameter is comprised between 46 and 93 nm (Figure 8b), which makes it difficult to determine the average diameter of the micelles from the cryo-TEM images. Such oval-shaped micelles have been rarely observed in the literature for self-assembled block copolymers. However, a similar morphology was already reported by Soto et al. [51], for example, using a non-trivial process. In their work, the authors used poly(ferrocenyldimethylsilane)-*b*-poly(2-vinylpyridine) (PFS-*b*-P2VP) micelles as seeds in the self-assembly of PFS-*b*-poly[bis (trifluoroethoxy)-phosphazene (PFS-*b*-PP) block copolymers.

### 3.5. Effect of the PNVCL-b-PCL Block Copolymer Concentration and Hydrophobic Block Length on the LCST of the Polymeric Micelles

**Figure 9:**
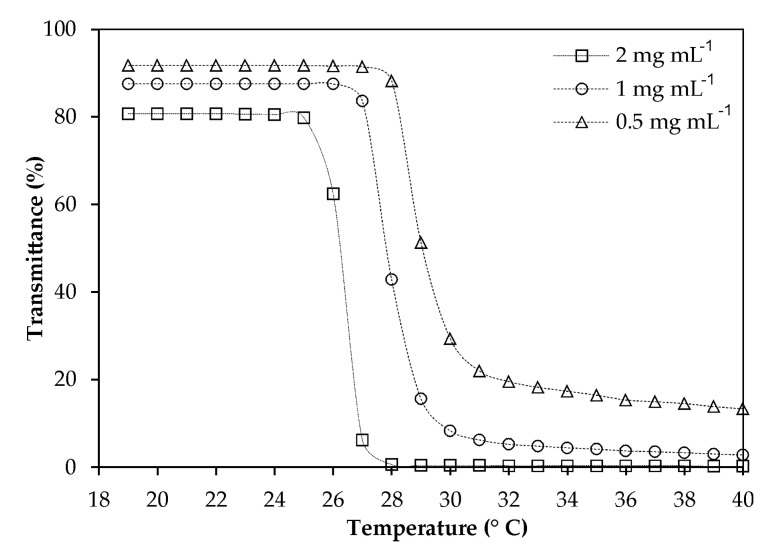
Effect of the concentration on the LCST of the PNVCL-*b*-PCL (1) block copolymer micelles.

In this study, the thermosensitive behavior of the PNVCL-*b*-PCL block copolymers micelles was tuned by changing the polymer concentration and the length of the hydrophobic PCL block. Figure 9 shows the temperature dependence of optical transmittance of micellar solutions of the copolymers (PNVCL-*b*-PCL (1), *M*_n NMR_ = 9629 g mol^−1^) prepared with three different concentrations.

As expected, the LCST of the PNVCL-*b*-PCL (1) block copolymer micelles increased from 26.4 to 29.2 °C as the copolymer concentration decreased from 2.0 to 0.5 mg mL^−1^, indicating that the LCST depends on the concentration. This result is consistent with the generally accepted LCST principle for dilute solutions, reported in the literature, which states that a higher water content enhances hydrogen-bonding interactions between water and the polymer chains, which requires more thermal energy to break the water structure, thereby resulting in an increase of the LCST [19].

**Figure 10:**
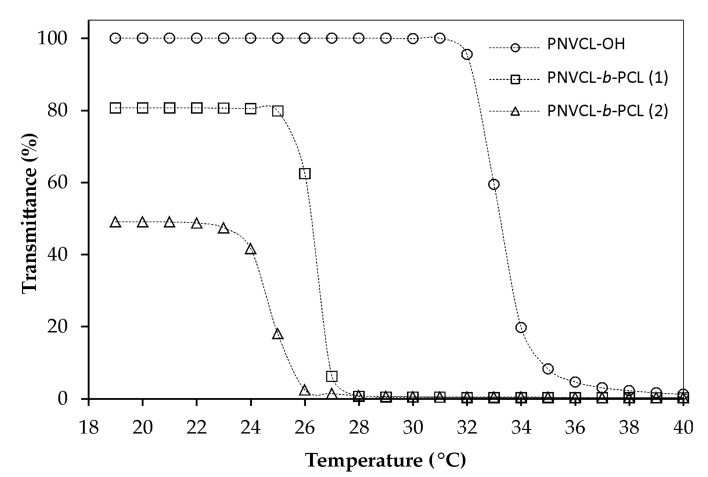
Effect of hydrophobic block length on the LCST of the PNVCL-*b*-PCL block copolymer micelles (C = 2 mg mL^−1^).

Figure 10 shows the effect of the molar mass of the PCL block on the LCST. The LCST was evaluated as 33.2, 26.4 and 24.7 °C for PNVCL–OH, PNVCL-*b*-PCL (1) and PNVCL-*b*-PCL (2), respectively. Considering the fact that PCL is hydrophobic, the PNVCL-*b*-PCL block copolymers are less hydrophilic than the PNVCL homopolymer. Therefore, the copolymer with the higher PCL block length (PNVCL-*b*-PCL (2)) is less hydrophilic than the PNVCL-*b*-PCL (1) copolymer resulting in lower LCST values, as expected. These results are in agreement with the literature [5,19,52,53], and show that the phase transition of the copolymers can be controlled within a temperature range by tuning the hydrophobicity of the (co)polymers.

### 3.6. Effect of Temperature on the Hydrodynamic Diameter of the Polymeric Micelles

**Figure 11:**
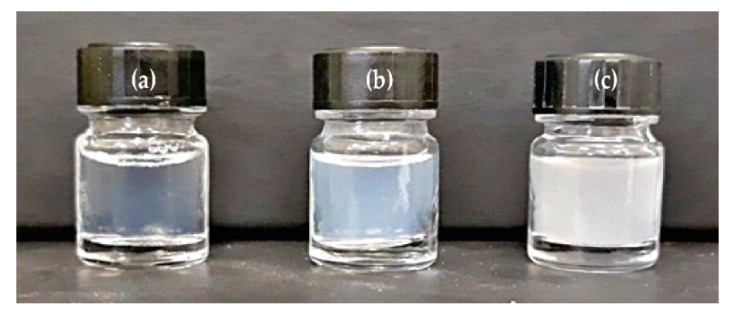
Visual aspects of the micellar solution of the PNVCL-*b*-PCL (1) block copolymer (C = 0.5 mg mL^−1^) at different temperatures, at around: (**a**) 19 °C, (**b**) 29 °C and (**c**) 40 °C.

Figure S6 (Supplementary Materials) shows the effect of the temperature on the diameter of the PNVCL-*b*-PCL (1) micelles in aqueous solution (*M*_n NMR_ = 9629 g mol^−1^, 2 mg mL^−1^). As shown in the Figure, when the solution temperature was below the LCST, the polymeric micelles existed individually and their diameter remained practically constant. However, at a certain temperature close to the LCST, intermicellar aggregation gave rise to the formation of large aggregates, associated with an abrupt increase in the hydrodynamic diameter, and a dramatic increase of the turbidity of the micellar solution. Next, we focused our attention on the polymeric micelles prepared at a lower concentration of 0.5 mg mL^−1^. Figure 11 shows digital photographs of the PNVCL-*b*-PCL (1) micellar solution at three different temperatures. As mentioned above, below the LCST of the PNVCL-*b*-PCL (1) micelles (previously determined at 29.2 °C), the PNVCL-*b*-PCL is amphiphilic, thus the solution was practically transparent (Figure 11a). When heated close to the LCST, the solution gradually turned into a semi-transparent bluish suspension (Figure 11b). Above the LCST, the solution became more turbid because, in this condition, the block copolymers are hydrophobic (Figure 11c). However, when cooled down below the LCST, a transparent solution could be again obtained, as shown below.

**Figure 12:**
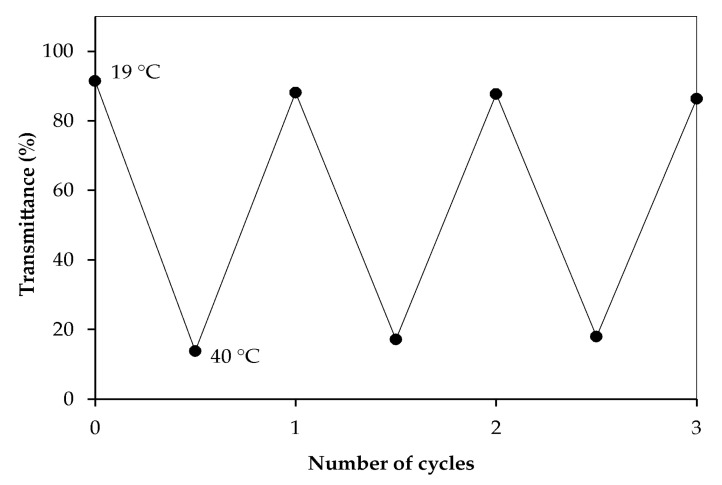
Transmittance cycling of the PNVCL-*b*-PCL (1) block copolymer micelle solution (0.5 mg mL^−1^) between 19 and 40 °C.

In order to confirm the reversible thermal phase transition of the PNVCL-*b*-PCL micelles, we have carried out some transmittance measurements. Figure 12 shows the transmittance cycling of the PNVCL-*b*-PCL (1) micelle solution between 19 °C (below the LCST) and 40 °C (above the LCST). The micellar solution became cloudy when the temperature increased from 19 to 40 °C, and reverted to a transparent solution when the temperature decreased from 40 to 19 °C showing almost the same initial transmittance value. This reversible change in the transmittance of the micellar solution with temperature reflects the different solvation of the PNVCL chains by water molecules at temperatures below and above the phase transition temperature. Besides, this result indicates that the micelles maintained their stability without precipitation during the cycle of temperature change. The aggregation and dispersion of such micelles in an aqueous solution have attracted considerable interest for targeted and controlled drug delivery [23,26].

## 4. Conclusions

In this study, a PNVCL homopolymer containing a hydroxyl end group (PNVCL–OH) and PNVCL-*b*-PCL block copolymers with two different PCL block lengths have been synthesized. The chemical structure of the PNVCL–OH obtained via NVCL RAFT/MADIX polymerization was confirmed by FTIR and ^1^H NMR analyses. In addition, the low dispersity value of the PNVCL–OH revealed a high efficiency of the *O*-ethyl S-4-(hydroxymethyl) benzyl carbonodithioate in controlling NVCL polymerization. SEC, FTIR, NMR and DSC analyses indicated successful synthesis of the PNVCL-*b*-PCL block copolymers by Ɛ-CL ring-opening polymerization using PNVCL–OH as the macroinitiator. Therefore, the results shown in this work proved the effectiveness of this synthetic route in the synthesis of amphiphilic PCL-*b*-PNVCL block copolymers composed of different PCL block lengths. The CMC values of the block copolymers decreased with increasing the length of the PCL segment. Properties of the micelles such as their size, morphology and thermosensitivity were investigated. The LCST of the PNVCL-*b*-PCL micelles was affected by the concentration and composition of the copolymer. The LCST decreased with increasing the copolymer concentration and the length of the PCL chain. The average diameter of the micelles determined by DLS increased with increasing the molar mass of the PCL bock, while cryo-TEM showed the formation of micelles with a pointed-oval-morphology with the hydrophobic PCL segments buried in the core and the hydrophilic PNVCL located at the surface. The PCL-*b*-PVCL micelles exhibited a reversible LCST phase transition during reversible cooling and heating cycles between 19 and 40 °C.

## Figures and Tables

**Table 1:** Related data on PNVCL–OH homopolymer obtained by NVCL Reversible Addition-Fragmentation Chain Transfer (RAFT)/macromolecular design by the interchange of xanthates (MADIX) polymerization.

Sample	Conv. ^a^ (%)	*M*_n theo._^b^ (g mol^−1^)	*M*_n NMR_^a^ (g mol^−1^)	*M*_n SEC_^c^ (g mol^−1^)	Đ ^c^
X-PNVCL–OH	34.0	7342	6379	3903	1.44
PNVCL–OH	-	^-^	6480	4177	1.43

^a^ Determined by ^1^H NMR. ^b^ *M*_n theo._ = ([NVCL]_0_/[CTA]_0_. MM_NVCL_. Conv) + MM_CTA–OH_. ^c^ Determined by SEC in THF using TEA (0.3% v/v) at 35 °C.

**Table 2:** Summary of the main characteristics of the PNVCL-*b*-PCL block copolymers obtained by ROP of ε-CL.

Reaction	Conv. ^c^ (%)	*M*_n theo._^d^ (g mol^−1^)	*M*_n NMR_^c^ (g mol^−1^)	*M*_n SEC_^e^ (g mol^−1^)	Ð ^e^	CMC ^f^ 10^−3^ (mg mL^−1^)	Z_av_^g^ (nm)	PdI ^g^
PNVCL-*b*-PCL (1) ^a^	92.4	10638	9629	6517	1.48	2.9	86	0.203
PNVCL-*b*-PCL (2) ^b^	97.6	14306	12719	9007	1.57	1.4	117	0.237

Reaction time = ^a^ 5 and ^b^ 6 h. ^c^ Determined by ^1^H NMR. ^d^ *M*_n theo_ = ([Ɛ-CL]_0_/[PNVCL–OH]_0_ × Conv. MM_Ɛ-CL_) + *M*_n theo. PNVCL–OH_, where *M*_n theo. PNVCL–OH_ ≈ *M*_n theo. X-PNVCL–OH_ = 7342 g mol^−1^. ^e^ Determined by SEC in THF and TEA (0.3% v/v) at 35 °C. ^f^ Determined by fluorescence measurements using pyrene as a probe. ^g^ Determined by DLS at 20 °C.

## Supplementary Materials

The following are available online at https://www.mdpi.com/2073-4360/12/6/1252/s1, Figure S1: ^1^H NMR spectrum of *O*-ethyl S-4-(hydroxymethyl) benzyl carbonodithioate, Figure S2: (a) Plot of monomer conversion vs. reaction time and (b) plots of the number average molar mass (*M*_n_, filled symbol) and dispersity (Ð, empty symbol) vs. monomer conversion for the polymerization of NVCL in 1.4-dioxane using [NVCL]:[CTA]:[AIBN] = 150:1:0.1 feed molar ratio at 70 °C, Figure S3: ^1^H NMR spectra of PNVCL homopolymer before (X-PNVCL–OH, a) and after (PNVCL–OH, b) reaction with AIBN, Figure S4: ^1^H NMR spectra of (a) PNVCL–OH and (b) PNVCL-*b*-PCL (1), Figure S5: Size distribution of the PNVCL-*b*-PCL micelles determined by DLS: (a) PNVCL-*b*-PCL (1) and (b) PNVCL-*b*-PCL (2) micelles, Figure S6: Temperature dependence of the size and optical transmittance of the PCL-*b*-PVCL (1) micelles (2 mg mL^−1^).
