# Supplementary material for: Synthesis and Self-Assembly of Poly(N-Vinylcaprolactam)-b-Poly(ε-Caprolactone) Block Copolymers via the Combination of RAFT/MADIX and Ring-Opening Polymerizations
Source: Polymers (Basel). 2020 May 30;12(6):1252. doi: 10.3390/polym12061252 (PMC7362203; doi:10.3390/polym12061252)
Supplement: Supplementary file 1 [file polymers-12-01252-s001.pdf]

polymers

MDPI

Synthesis and self-assembly of poly(*N*-vinylcaprolactam)-*b*-poly(ε-caprolactone) block copolymers via the combination of RAFT/MADIX and ring-opening polymerizations

Rodolfo M. Moraes,[1] Layde T. Carvalho,[1] Gizelda M. Alves,[1] Simone F. Medeiros,[1] Elodie Bourgeat-Lami,[2*] and Amilton M. Santos [1,*]

SUPPLEMENTARY MATERIALS

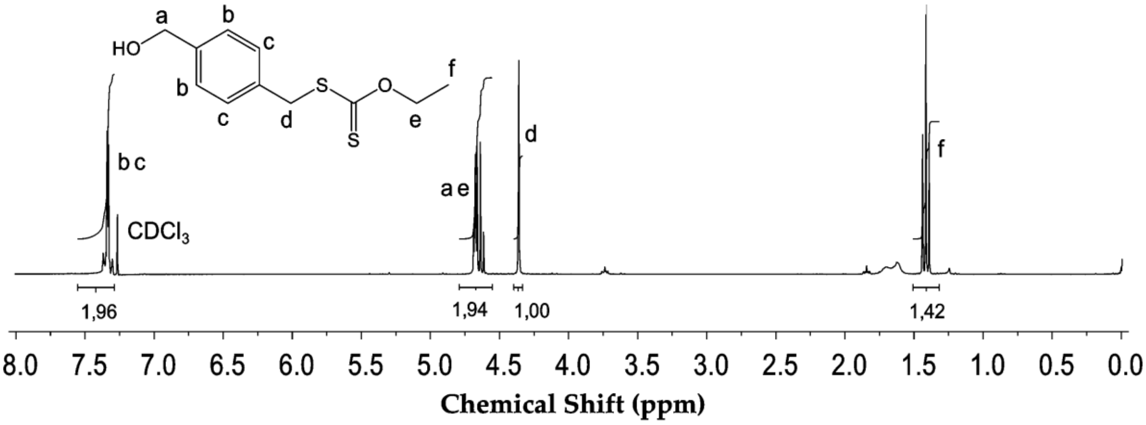

Figure S1. ^{1}H NMR spectrum of *O*-ethyl S-4-(hydroxymethyl)benzyl carbonodithioate.

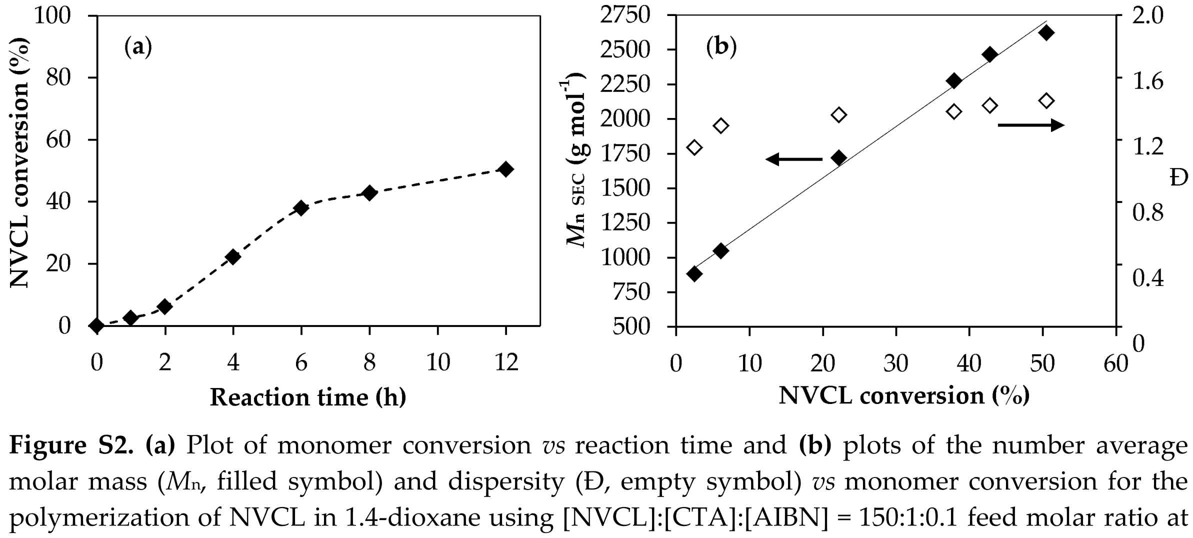

Figure S2. (a) Plot of monomer conversion *vs* reaction time and **(b)** plots of the number average molar mass (M_n, filled symbol) and dispersity (Đ, empty symbol) *vs* monomer conversion for the polymerization of NVCL in 1.4-dioxane using [NVCL]:[CTA]:[AIBN] = 150:1:0.1 feed molar ratio at 70 °C. The solid line is the fit line for M_n determined by HPLC.

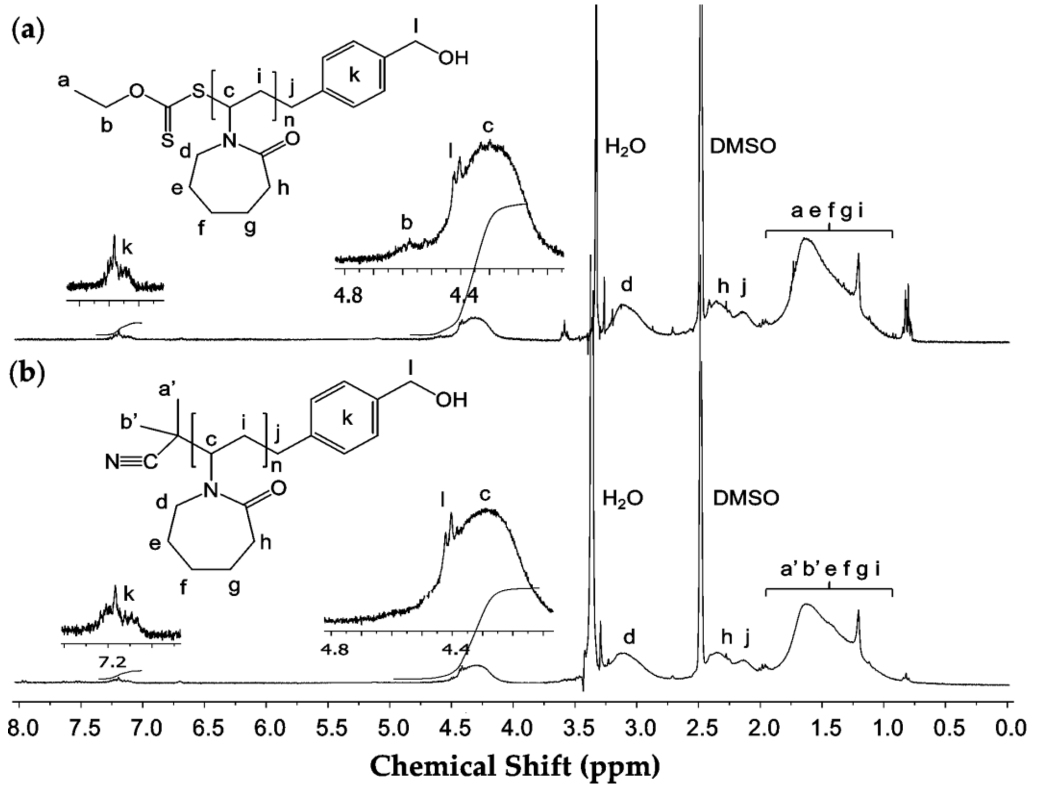

Figure S3. ^{1}H NMR spectra of PNVCL homopolymer before (X-PNVCL-OH, **a**) and after (PNVCL-OH, **b**) reaction with AIBN.

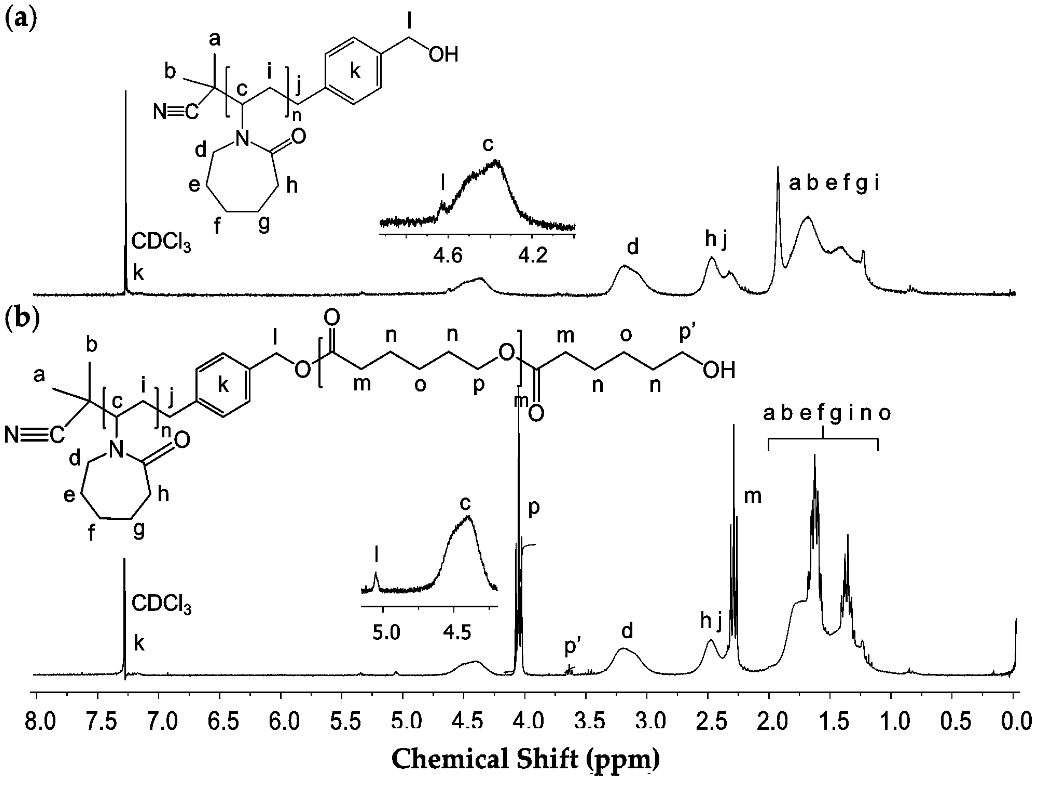

Figure S4. ^{1}H NMR spectra of (**a**) PNVCL-OH and (**b**) PNVCL-*b*-PCL (1).

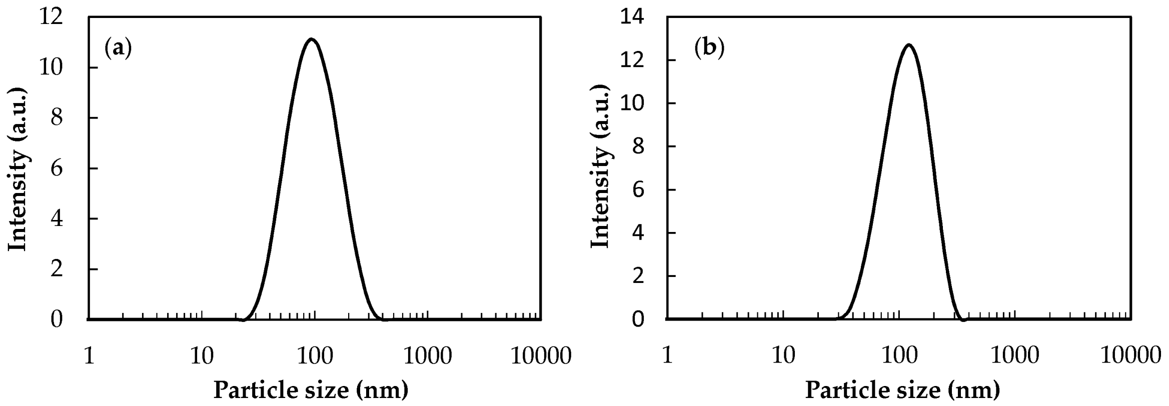

Figure S5. Size distribution of the PNVCL-*b*-PCL micelles determined by DLS: (**a**) PNVCL-*b*-PCL (1) and (**b**) PNVCL-*b*-PCL (2) micelles.

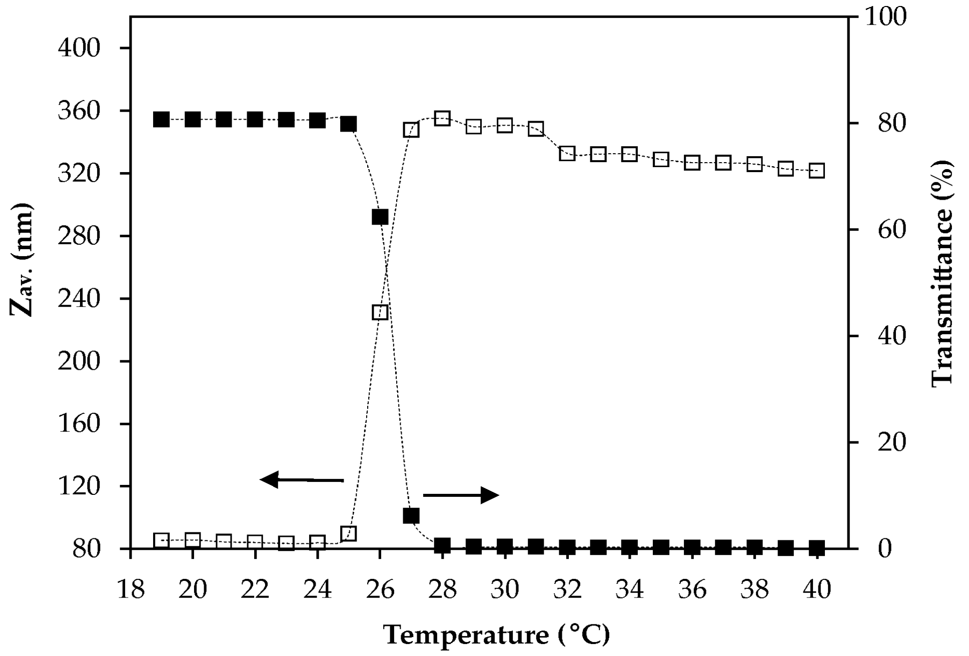

Figure S6. Temperature dependence of the size and optical transmittance of the PCL-*b*-PVCL (1) micelles (2 mg mL^{-1}).

© 2020 by the authors. Submitted for possible open access publication under the terms and conditions of the Creative Commons Attribution (CC BY) license (http://creativecommons.org/licenses/by/4.0/).
